# Cytotoxicity, Epidermal Barrier Function and Cytokine Evaluation after Antiseptic Treatment in Bioengineered Autologous Skin Substitute

**DOI:** 10.3390/biomedicines10061453

**Published:** 2022-06-19

**Authors:** Marta García-Valdivia, María I. Quiñones-Vico, Laura Ortega-Llamas, Ana Fernández-González, Ana Ubago-Rodríguez, Raquel Sanabria-de la Torre, Salvador Arias-Santiago

**Affiliations:** 1 Cell Production and Tissue Engineering Unit, Virgen de las Nieves University Hospital, 18014 Granada, Spain; martagv9495@gmail.com (M.G.-V.); maribelqv@ugr.es (M.I.Q.-V.); laura.ortega.llamas@gmail.com (L.O.-L.); aur@ugr.es (A.U.-R.); raquelsanabriadlt@gmail.com (R.S.-d.l.T.); salvadorarias@ugr.es (S.A.-S.); 2 Biosanitary Institute of Granada (ibs.GRANADA), 18014 Granada, Spain; 3 Andalusian Network of Design and Translation of Advanced Therapies, 41092 Seville, Spain; 4 Dermatology Department, School of Medicine, University of Granada, 18014 Granada, Spain; 5 Dermatology Department, Virgen de las Nieves University Hospital, 18014 Granada, Spain

**Keywords:** bioengineered autologous skin substitute, cell viability, cytokine secretion, drug development, epidermal barrier function, in vitro model, regenerative medicine, tissue engineering

## Abstract

Bioengineered autologous skin substitutes (BASS) technology is an emerging field for skin burn therapy. However, further studies on BASS characterization, viability against standard procedures for wound healing, and protocol optimization are necessary for the improvement of BASS technology for clinical use. The aim of this study is to evaluate the effect of common antiseptics for clinical use in BASS, focusing on cell viability, inflammatory cytokine pattern, and epithelium and skin barrier integrity, in order to establish the most adequate treatment for wound care after BASS grafting. Human keratinocytes (hKT) and dermal fibroblasts (hDF) were isolated from foreskin samples and integrated into hyaluronic acid-based BASS. The following antiseptics were applied every 48 h: ethanol (70%), chlorhexidine digluconate (1%), sodium hypochlorite (0.02%), povidone iodine (100 mg/mL), and polyhexanide (0.1%), during a follow-up of 16 days. Sodium hypochlorite was the only treatment that showed a high cell viability percentage throughout the evaluation time compared to other antiseptic treatments, as well as a similar cytokine secretion pattern as control BASS. No significant differences were found regarding epidermal barrier function. These findings point towards sodium hypochlorite being the least aggressive antiseptic treatment for BASS post-transplantation wound care.

## 1. Introduction

Burns are one of the most common causes of injury worldwide, affecting over 30 million people per year. According to the World Health Organization (WHO), burn wounds account for an average of 180,000 deaths annually, the majority of these occurring in low- and middle-income countries [1,2].

Non-fatal burns are a leading cause of morbidity and are related to prolonged hospitalization. They are the direct cause of chronic pain, disfigurement and disability, oftentimes leading to social stigma and discrimination [1,2]. Burn care is related to a high hospitalization cost, due to long hospital stays, specialized dressing, multiple surgeries and the need of intensive care in severe cases [2].

The skin is one of the most complex organs in the human body, comprising 15% of the total adult body weight, and it constitutes the barrier between the internal and external environment [3]. It is responsible for the maintenance of the homeostasis, thermoregulation and hydration of the living body, and it provides protection against external harmful agents and pathogens [4].

Due to the presence of stem cells, the skin has the ability to stimulate self-regeneration. However, a significant loss of skin tissue from second- or third-degree burns or deep injuries can compromise its self-renewal properties, also leaving the body unprotected against infection. Additionally, these injuries often lead to the formation of chronic wounds that further hinder the survival of the patient [3,5]. Therefore, clinical intervention is crucial to provide an ideal environment for healing these wounds.

The usual clinical protocol for burn treatment includes a thorough cleansing and debridement of blisters, as well as the application of wound dressings to minimize contact with outer pathogens and provide a humid environment to potentiate the healing process [2]. In wounds that require further intervention, different strategies using skin substitutes have been developed in the recent years. These substitutes have the function of preventing wound infection by covering the injured site and reduce the patient’s pain, as well as promoting wound healing by releasing cytokines and growth factors at the wound site, and replacing the missing skin to restore its functionality [3,6,7].

Autologous skin grafts, allografts, and xenografts are categorized as conventional skin substitutes for wound healing. Although these strategies have the advantage of maintaining the skin structure, they also have grave disadvantages, such as a limited availability in the case of autologous skin drafts or allografts, and the risk of rejection and viral transmission associated with allografts and xenografts [3,7].

New approaches based on tissue engineering include cell suspensions and cocultures, acellular skin substitutes and allogenic commercial skin substitutes [3]. These strategies, while they solve the problem of availability in conventional treatments, also have several disadvantages. In the case of cell suspensions and cocultures, they fail to achieve complete biological function, and they have risk of rejection if the cells used in the treatment are allogenic [8]. Rejection risk is also a problem associated with commercial allogenic skin substitutes. Acellular matrixes lack of a cellular component limiting the skin regeneration and restricting their use to only temporary wound coverage [3,8].

Another skin substitute approach, which we will be focusing on, is the use of Bioengineered Autologous Skin Substitutes (BASS). BASS is an autologous graft consisting of two layers; a basal layer composed of fibroblasts immersed in a scaffold mimicking the extracellular matrix (ECM) to substitute the dermal stroma, and an epithelium of keratinocytes seeded on top [3,5,8]. The scaffold has the function of anchoring the cells to the graft. It has a role in cell survival and proliferation, participates in the control of homeostasis and provides an optimal environment for wound healing [3,8]. Hyaluronic acid (HA) is one of the most widely used ECM components in wound dressings, stimulating cellular proliferation, cellular migration, and angiogenesis [9].

Once a skin substitute is applied onto the patient’s wound, cutaneous treatment with antiseptics is crucial to prevent infection of the wound. However, there is little information about the impact of these treatments on the viability of the cells in BASS [8,10]. There are few studies analyzing the effect of antiseptic treatment in human keratinocytes (hKT) and dermal fibroblasts (hDF). However, they all report relevant cytotoxicity related to antiseptic treatment in cell culture and wound dressing [10,11].

Therefore, the objective of this work is to perform an exhaustive evaluation on the effect of the antiseptics used in clinic on three-dimensional, scaffold-based substitutes such as BASS. More concretely, the effect of antiseptic treatment on cell viability, cytokine pattern, epithelium integrity, and barrier function were studied, in order to determine which protocol is the ideal to follow in burn care in patients using BASS.

## 2. Materials and Methods

### 2.1. Cell Isolation and Culture

hKT and hDF were obtained from foreskin samples (9 cm^2^) obtained from plastic or dermatological and urological surgery surplus, with the patients’ informed consent in compliance with the requirements for donation of human cells and tissues (Royal Decree-Law 9/2014, of July 4). The study was approved by the Provincial Ethics Committee of Granada (Spain). Skin samples were washed and transported in Dulbecco’s phosphate-buffered saline (DPBS, Sigma Aldrich, St Louis, MO, USA). In a laminar flow cabin, the dermis and epidermis were mechanically separated and processed with scalpels and forceps, while the hypodermis was discarded.

The dermis was incubated in a 2 mg/mL solution of type I collagenase (Gibco, Thermo Fisher Scientific, West Sacramento, CA, USA) for 18–24 h, and neutralized with dermal fibroblasts culture medium (DFM, DMEM 10% fetal bovine serum (FBS), 2% glutamine, 0.1% gentamicin). The epidermis was incubated with TrypLE Select 10× (Gibco, Thermo Fisher Scientific, West Sacramento, CA, USA) for 8–10 cycles of 15 min each, and neutralized with specific medium for keratinocyte culture (KTM, DMEM 10% FBS, 2% glutamine, 96 µg/mL gentamicin, 0.4 µg/mL hydrocortisone, 5 µg/mL insulin, 1.4 µg/mL triiodo thyronine, 24 µg/mL adenine, 0.01 µg/mL epidermal growth factor, 1.25 µg/mL amphotericin B). Cells were centrifuged at 1000 rpm for 10 min, and the supernatants were discarded. Cell counting and viability were determined with Türk (Sigma Aldrich, St Louis, MO, USA) and trypan blue (Sigma Aldrich, St Louis, MO, USA) solutions, respectively.

hDF were seeded at a density of 80,000–100,000 cells/cm^2^ at 37 °C and 5% CO_2_. For hKT culture, 3T3 murine fibroblasts (3T3 Swiss Albino, ATCC, CCL-92, 60770553) were used as a feeder [12]; 3T3 were irradiated sub-lethally (50 Gy) and seeded at a density of 40,000 cells/cm^2^ a day before sample processing, while hKT were seeded at a density of 20,000–40,000 cells/cm^2^ over the layer of irradiated 3T3 cells (3T3i). (37 °C, 5% CO_2_).

### 2.2. BASS Manufacturing

The BASS used in this study consists of a bi-layer cultured scaffold made up of a hyaluronic acid matrix containing fibroblasts covered by an epithelium of keratinocytes. The hyaluronic acid matrix was made by mixing human plasma, DFM-containing fibroblasts, hyaluronic acid (Hyalone, Fidia Pharma, Abano Terme, Italy), calcium chloride (B Braun Medical, Barcelona, Spain, 10 mg/mL), and tranexamic acid (Amchafibrin, Rottapham, Spain). After 24 h, hKT were seeded to constitute the upper layer of the BASS.

**Figure 1:**
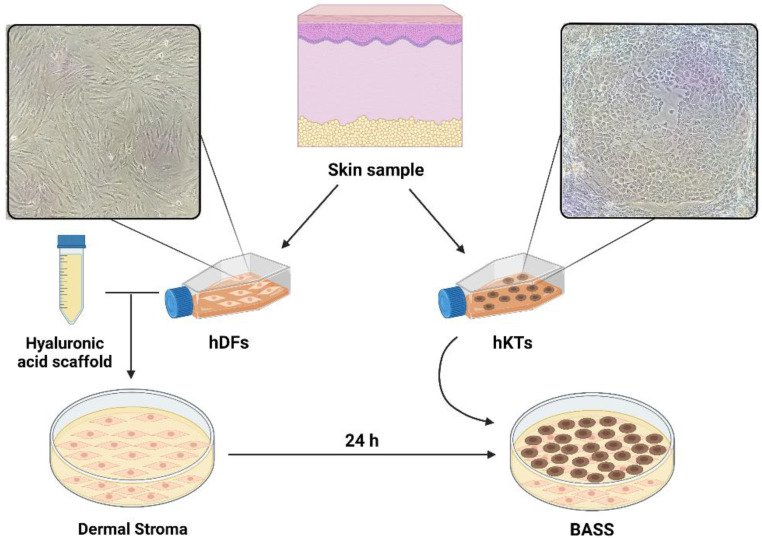
Schematic representation of cell culture and BASS manufacturing process. Created with BioRender.com. Human dermal fibroblasts (hDFs), human keratinocytes (hKTs), bioengineered autologous skin substitute (BASS).

BASS were incubated for 6 days before treatment started. Figure 1 represents the cell culture and BASS manufacturing process.

### 2.3. Antiseptic Treatment

BASS were treated with an assortment of antiseptics every 48 h for 16 days, while controls BASS were left untreated. The antiseptics used were the following: ethanol 70% (Betamadrileño S.L., Madrid, Spain), chlorhexidine digluconate 1% (HiBiSCRUB^®^, Molnlycke Health Care AB, Madrid, Spain), sodium hypochlorite 0.02% (Microdacyn, Sonoma Pharmaceuticals, West Sacramento, CA, USA), povidone iodine 100 mg/mL (Povidona, LAINCO, S.A., Barcelona, Spain), and polyhexanide 0.1% (Prontosan, B Braun Medical, Barcelona, Spain). The antiseptics concentration and treatment protocols duration used in this study were according to directions of use; treatment duration for chlorhexidine digluconate and povidone iodine groups were 3 min, while ethanol, sodium hypochlorite and polyhexanide groups were treated for 15 min.

### 2.4. BASS Test

Several studies for BASS analysis were performed on day 6, 9, 13, and 16 after BASS manufacturing.

#### 2.4.1. Viability Test

Cell viability and antiseptic cytotoxicity was tested with LIVE/DEAD^®^ Cell Viability Assay (Thermo Fisher Scientific, West Sacramento, CA, USA), a fluorescence-based staining of calcein AM (green fluorescence, Ex/Em 494/517 nm) and ethidium homodimer-1 (red fluorescence, Ex/Em 517/617 nm), which allows a colorimetric discrimination of the live and dead cell population.

BASS were washed with DPBS, and after adding the staining solution, they were incubated at room temperature and darkness for 30 min. After, the staining solution was discarded. The skin substitutes were washed with DPBS and their fluorescence measurement using a Leica DM2000 microscope. The images were analyzed using the software ImageJ v1.47. (Bethesda, MA, USA)

#### 2.4.2. ELISA Assay

In order to perform the ELISA assay, BASS supernatants of each treatment group were collected on day 6, 9, and 16. The concentration levels of interleukin 10 (IL-10, Sigma Aldrich, St Louis, MO, USA) an anti-inflammatory cytokine [13], basic fibroblast growth factor (bFGF, Sigma Aldrich, St Louis, MO, USA), a growth factor which promotes angiogenesis and the synthesis of extracellular matrix [14], and tumor necrosis factor alpha (TNF-α, Sigma Aldrich, St Louis, MO, USA), a pro-inflammatory cytokine [14] were quantified in BASS supernatants by ELISA following the manufacturer’s instructions. Absorbance levels were measured at 450 nm, and protein concentration was measured using a standard plot.

#### 2.4.3. Epidermal Barrier Function Evaluation

The epidermal barrier function evaluation by measuring the trans-epidermal water loss (TEWL) is another form to assess BASS functionality. TEWL measures the condensed water diffused through the skin surface per unit of time. Damage in the skin barrier will result in a higher water loss, translating into an increase of TEWL, while an intact skin barrier will retain water more efficiently, resulting in a lower TEWL [5].

TEWL was measured using a Tewameter^®^ VT 310 in vitro probe, and a Tewameter^®^ TM 300 in vivo probe (Courage + Khazaka Electronic, Köln, Germany). The Tewameter^®^ probe measures the water evaporation density of a surface through two sensors (temperature and relative humidity) inside a hollow cylinder. The measurement follows Adolf Flick’s law of diffusion:

(1) $$\frac{dm}{dt}=\frac{-D\times A\times dp}{dx}$$

where “m” represents the water transported (g) and “t” represents the time (h), “D” is the diffusion constant (0.0877 g/m (h mmHg)), “A” represents the surface (m^2^), “p” is the atmosphere vapor pressure (mm Hg), and “x” is the distance from the surface to the measurement point (m).

Before starting the measurement, dehydration by controlled pressure was performed using a glass disc of 85 g for 1 min in order to improve their biomechanical properties [15].

Additionally, 100 µL of Vaseline (Reig Jofre Laboratoy S.A., Barcelona, Spain) was spread over the BASS surface in order to evaluate the effect of Vaseline usage in the epidermal barrier function. Once applied, TEWL measures were taken at 0 min and 15 min using the in vitro probe. MPA software (Multi Probe Adapter, Courage + Khazaka Electronic, Köln, Germany) was used to analyze the data.

### 2.5. Statistical Analysis

For statistical analysis, the software GraphPad Prism (GraphPad Software, Inc., La Jolla, CA, USA) was used. All data were expressed as mean ± standard error of the mean (SEM). Statistical significance was calculated by two-way ANOVA in the case of three variables and one-way ANOVA in case of two variables.

Once ANOVA was performed, a post hoc analysis was performed with Tukey’s test for all factors to evaluate the degree of significance when comparing the factor classes. Values of *p* ≤ 0.05 were considered statistically significant. All experiments were performed in triplicate (*n* = 3).

## 3. Results

### 3.1. Antiseptic Treatment Affects Cell Viability

**Figure 2:**
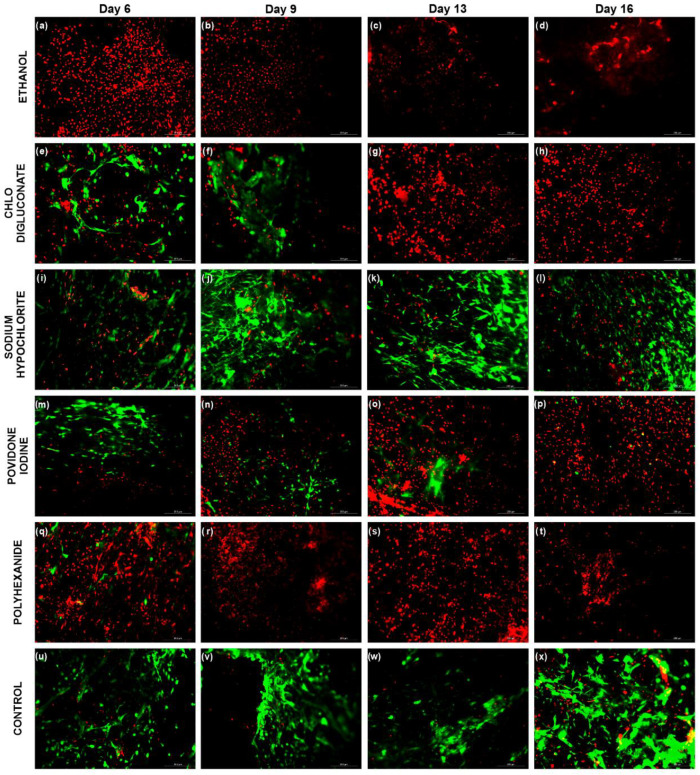
LIVE/DEAD^®^ fluorescence images of BASS at days 6, 9, 13, and 16. (**a**–**d**) BASS after ethanol (70%) treatment. (**e**–**h**) BASS after chlorhexidine digluconate (1%) treatment. (**i**–**l**) BASS after sodium hypochlorite (0.02%) treatment. (**m**–**p**) BASS after povidone iodine (100 mg/mL) treatment. (**q**–**t**) BASS after polyhexanide (0.01%) treatment. (**u**–**x**) control BASS. Live cells are represented in green, and dead cells in red. *n* = 3. Magnification 10×.

**Figure 3:**
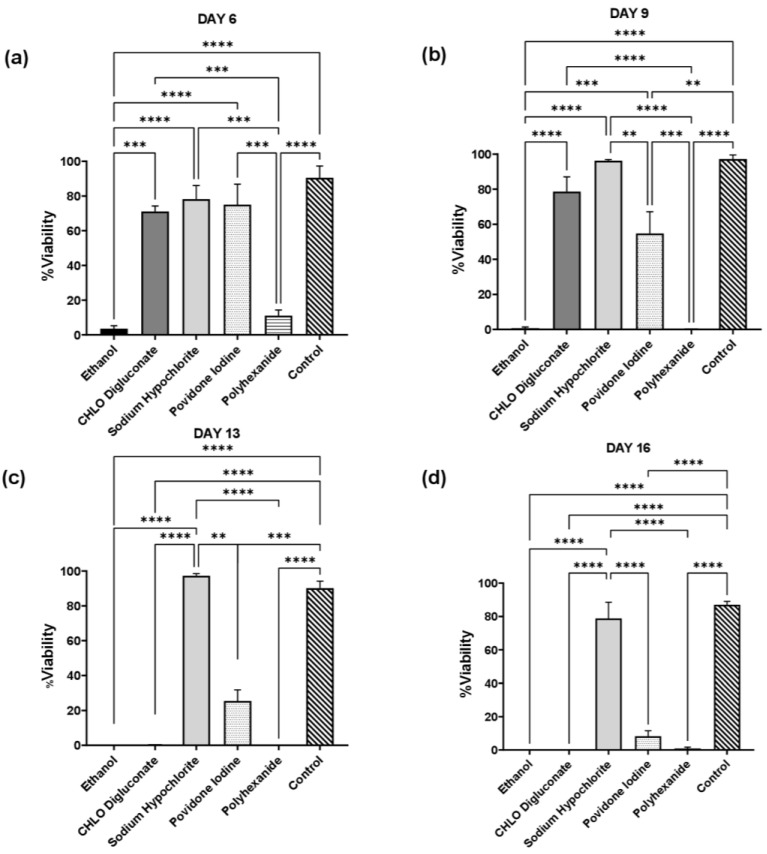
Statistical analysis of cell viability test for each antiseptic treatment group and control. Analysis in day 6 (**a**), day 9 (**b**), day 13 (**c**), and day 16 (**d**). *n*=3. Significances were represented as ** *p* < 0.01; *** *p* < 0.001; **** *p* < 0.0001.

The impact of antiseptic treatment in BASS cell viability was represented in Figure 2 and supplementary Table S1. The statistical analysis (Figure 3) revealed significant differences between the different treatment groups and control in each day of evaluation. On day 6 (Figure 3a), cell viability on BASS treated with ethanol and polyhexanide was significantly lower compared to control (*p* = 0.0001 in both cases) and other treatments (chlorhexidine digluconate *p* = 0.0001 and *p* = 0.0004; sodium hypochlorite *p* < 0.0001 and *p* = 0.0001; povidone iodine *p* < 0.0001 and *p* = 0.0002 respectively), while chlorhexidine digluconate, sodium hypochlorite and povidone iodine groups showed no significant differences in cell viability compared to control or between each other (*p* > 0.05).

On day 9 (Figure 3b), there was a significant reduction in cell viability in the povidone iodine treatment group compared to control (*p* = 0.0042) and sodium hypochlorite treatment (*p* = 0.005), while on day 13 (Figure 3c) there was a drastic decrease of cell viability on the chlorhexidine digluconate treatment group, resulting in statistical differences compared to control and sodium hypochlorite treatment (*p* <0.0001 in both cases). In the case of ethanol, povidone iodine and polyhexanide, they continued to show a significantly lower cell viability percentage compared to control and sodium hypochlorite treatment (*p* <0.0001 in the three treatment groups compared to control, and *p* <0.0001, *p* = 0.0029 and *p* <0.0001 compared to the sodium hypochlorite treatment).

Finally, on day 16 (Figure 3d), cell viability in ethanol, chlorhexidine digluconate, povidone iodine and polyhexanide treatment groups remained significantly lower compared to control and sodium hypochlorite treatment groups (*p* <0.0001), while the sodium hypochlorite treatment BASS still showed no significant differences in cell viability compared to control (*p* = 0.7884). Therefore, in terms of cell viability, sodium hypochlorite treatment was the only treatment that did not affect cell viability.

### 3.2. Cytokine Secretion Is Altered after Antiseptic Treatment

**Figure 4:**
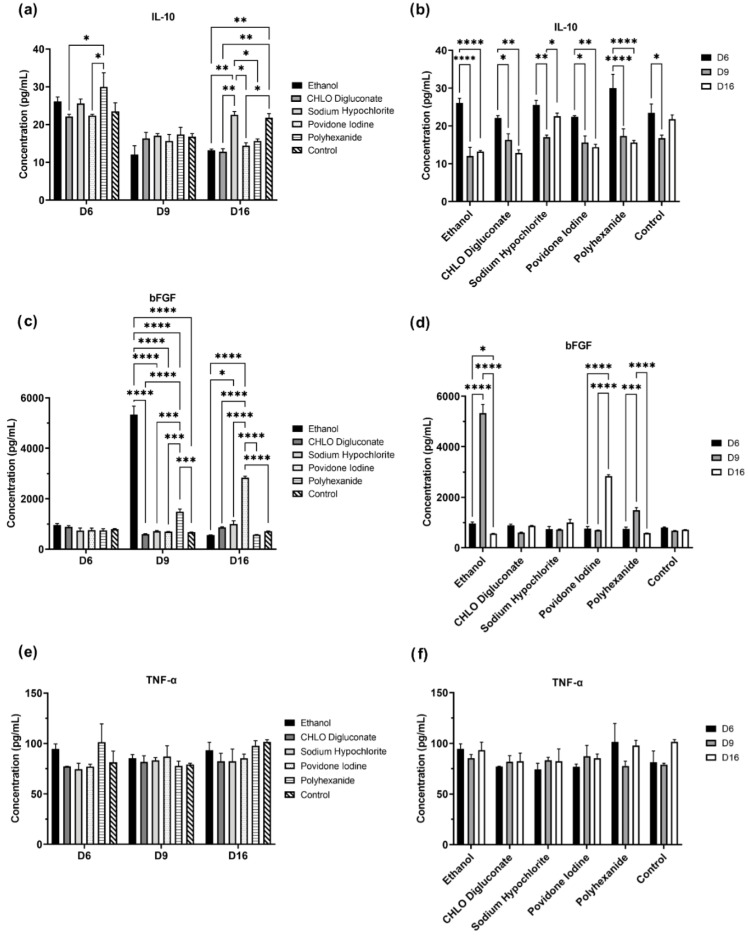
Statistical analysis of cytokine concentration levels in BASS supernatant on days 6, 9, and 16. Corresponding to IL-10 (**a**,**b**), bFGF (**c**,**d**), and TNF-α (**e**,**f**). *n*=3. Significances were represented as * *p* < 0.05; ** *p* < 0.01; *** *p* < 0.001; **** *p* < 0.0001.

In order to evaluate the secretion pattern of BASS after treatment with antiseptics, we studied the concentration of three cytokines, IL-10, bFGF, and TNF-α, in BASS supernatants at day 6, 9, and 16 after BASS treatment. Cytokine concentrations obtained were represented in supplementary Tables S2–S4, and the statistical analysis was illustrated in Figure 4.

In the analysis of IL-10 (Figure 4 a,b), significant differences were observed between antiseptic treatment groups on the different days of evaluation. On day 6 (Figure 4a), the concentration levels of IL-10 were significantly higher in the polyhexanide treatment group compared to chlorhexidine digluconate and povidone iodine treated BASS (*p* = 0.0185 and *p*= 0.0242, respectively). On day 9, no significant differences were observed in the different treatment groups and the control. However, on day 16, IL-10 levels on sodium hypochlorite treatment group and control were significantly higher compared to other treatments (in the case of sodium hypochlorite treatment, *p* = 0.0041, *p* = 0.0028, *p* = 0.0135, and *p* = 0.0433 compared to ethanol, chlorhexidine digluconate, povidone iodine, and polyhexanide respectively; in the case of control BASS, *p* = 0.0087 compared to ethanol, *p* = 0.0060 compared to chlorhexidine digluconate, and *p* = 0.0283 compared to povidone iodine).

Focusing on IL-10 concentration levels for each treatment throughout the evaluation period (Figure 4b), significant differences were also found. IL-10 levels in ethanol, chlorhexidine digluconate, povidone iodine and polyhexanide were significantly higher on day 6 compared to day 9 and 16 (*p* <0.0001 in both cases in ethanol; *p* = 0.0364 compared to day 9 and *p* = 0.0011 compared day 16 in the case of chlorhexidine digluconate; *p* = 0.0141 on day 9 and *p* = 0.0041 on day 16 in povidone iodine, and *p* <0.0001 in both cases in polyhexanide treatment group). In the case of sodium hypochlorite, there is a significant decrease of the concentration of IL-10 from day 6 to day 9 (*p* = 0.0022), which later increases again on day 16 (*p* = 0.0454 compared to day 9). Regarding the control group, there is a spike of IL-10 concentration levels on day 6, which later lowers on day 9 (*p* = 0.0151) and is maintained until the end of the evaluation period.

In the analysis of bFGF, we found no differences between treatments on day 6 (Figure 4c, d). However, we observed a high increase on bFGF levels in the ethanol and polyhexanide treatment groups on day 9, compared to the other treatments and control (in the case of ethanol, *p* <0.0001 in all comparisons; and in the case of polyhexanide, *p* <0.0001 compared to ethanol and chlorhexidine digluconate, and *p* = 0.0003, *p* = 0.0002, and *p* = 0.0001, compared to sodium hypochlorite, povidone iodine and control) (Figure 4c). On day 16, the concentration levels of bFGF diminished significantly on ethanol and polyhexanide treatment groups (*p* <0.0001 in both cases) (Figure 4d), and increased significantly in the povidone iodine treatment group (*p* <0.0001 compared to other treatments and control, and *p* <0.0001 compared to povidone iodine bFGF levels on day 6 and 9).

Finally, no significant differences in TNF-α concentration levels were observed in any of the antiseptic treatment groups, nor within the same treatment throughout the different days of evaluation (Figure 4e, f).

In summary, sodium hypochlorite was the only treatment that showed a similar cytokine secretion pattern as control BASS.

### 3.3. Trans-Epidermal Water Loss (TEWL) Analysis in BASS

#### 3.3.1. Antiseptic Treatment did Not Significantly Affect Trans-Epidermal Water Loss

**Figure 5:**
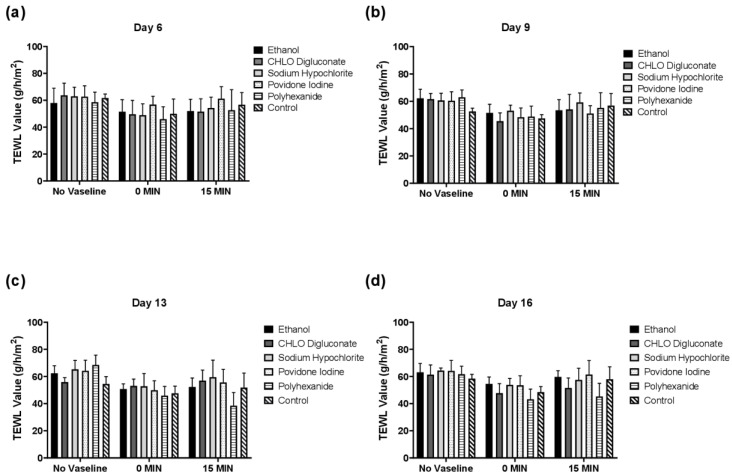
Statistical analysis of TEWL measurements of BASS treatment and control groups on day 6 (**a**), day 9 (**b**), day 13 (**c**), and day 16 (**d**). Graphic shows measures of BASS without Vaseline, with Vaseline at t = 0 min, and 15 min post-application. *n* = 3.

As shown in Figure 5, no significant differences in TEWL values were observed in any of the antiseptic treatment groups nor in the control group throughout the evaluation time (*p* > 0.05). Furthermore, no significant differences were shown after the application of Vaseline nor 15 min post-application.

#### 3.3.2. Vaseline Application Shows a Trans-Epidermal Water Loss Reduction Tendency in All BASS Study Groups

**Figure 6:**
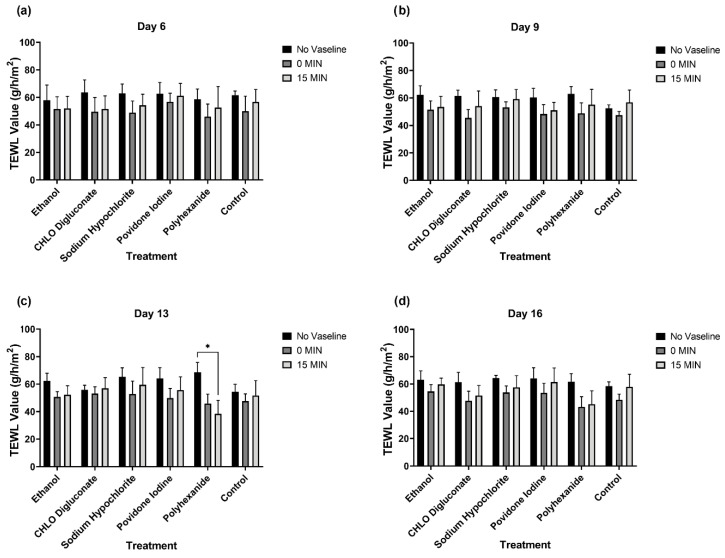
Statistical analysis of TEWL values on BASS without Vaseline, Vaseline at t = 0 min, and 15 min post-application on treatment and control BASS groups. Data organized into day 6 (**a**), day 9 (**b**), day 13 (**c**), and day 16 (**d**). *n* = 3.

Once the effect of antiseptic treatments in TEWL values in BASS scaffolds was analyzed, we studied the effect of Vaseline application in these constructs, measured immediately after Vaseline application (t = 0 min) and 15 min post-application. As represented in Figure 6, there were no significant differences in TEWL values in any of the measurement days (*p* > 0.05). However, a TEWL value reduction tendency was observed in all study groups after Vaseline application, which was either maintained or showed an insignificant increase 15 min post-application. The only antiseptic treatment that showed a decrease in TEWL value 15 min after Vaseline application was polyhexanide on day 13 (Figure 6c), showing a significant decrease in TEWL value compared to TEWL prior to Vaseline application (*p* = 0.0219).

#### 3.3.3. In Vitro and In Vivo Probes Failed to Detect Differences in Trans-Epidermal Water Loss in BASS

**Figure 7:**
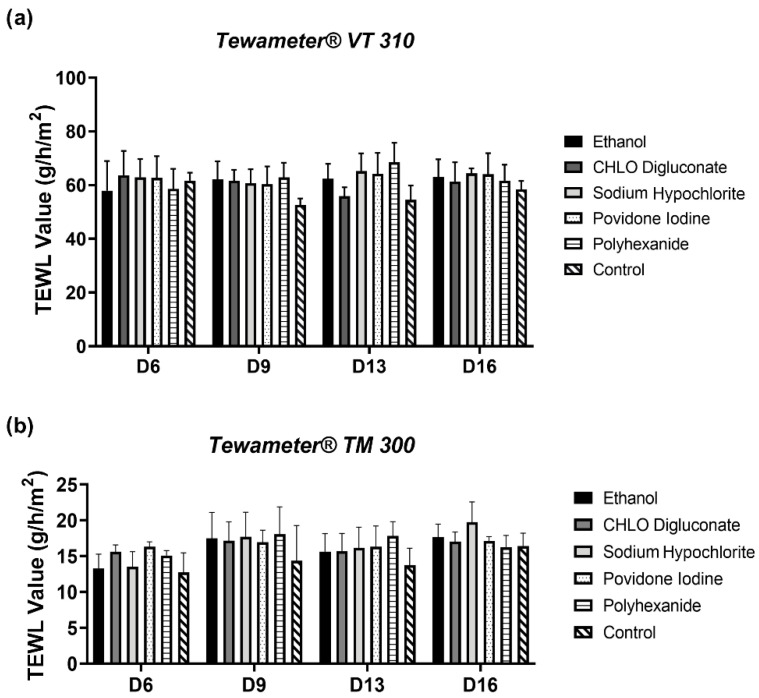
Statistical analysis of TEWL measurements on days 6, 9, 13, and 16 using an in vitro (Tewameter^®^ VT 310) (**a**) and in vivo (Tewameter^®^ TM 300) probe (**b**). *n* = 3.

In order to further study the ideal procedure to measure TEWL in BASS, two probes, Tewameter^®^ VT 310 in vitro probe and Tewameter^®^ TM 300 in vivo probe were evaluated. However, both the in vitro and the in vivo probe failed to show any significant differences in any of the antiseptic treatment groups compared to control or between treatments (*p* > 0.05) (Figure 7).

**Figure 8:**
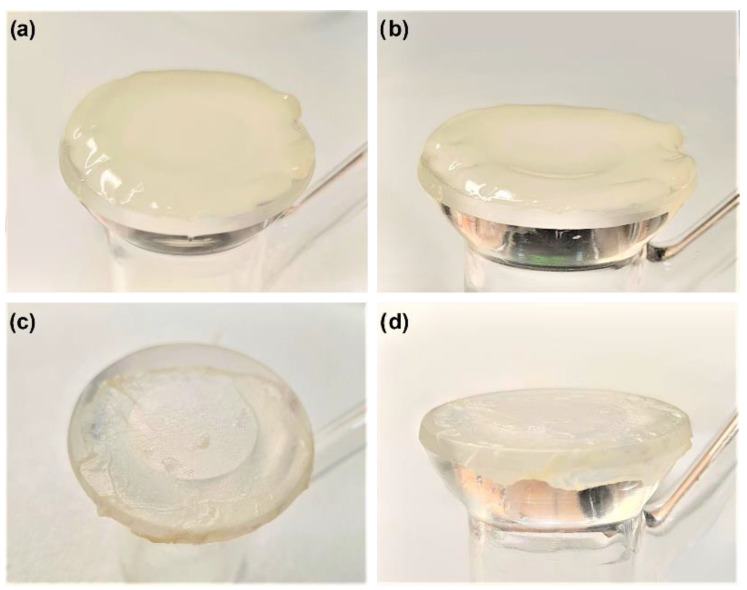
Pictures of control BASS (**a**,**b**) and treated BASS (**c**,**d**) on day 16 of BASS evaluation. Pictures taken from above (**a**,**c**) and the side (**b**,**d**) for better comparison. Treated BASS is thinner and more translucent compared to control BASS.

In summary, no significant differences were found in TEWL values after antiseptic treatment. Remarkably, dryness and thickness differences were found between BASS study groups post-dehydration (Figure 8).

## 4. Discussion

The translation of BASS from preclinical investigation to its use in clinic as an alternative for burn wound treatment is still in progress. Several studies using BASS have been carried out in patients sustaining burns on an average of 70% of the total body surface area and the results showed a significant clinical improvement, with an 80% survival rate and histological evidence of re-epithelialization in the burned area, as well as a homeostatic resemblance to healthy skin [5,16,17].

Nevertheless, even with the recent advancements already underway in BASS technology, there is still much more to study in order to characterize and standardize burn treatment with BASS technology. There is a previous study that performed an initial approach on the effect of antiseptics and antibiotics on this skin substitute [8]. Specifically, Quiñones-Vico et al. showed that the use of antiseptics can affect the cell viability and epithelium integrity of these substitutes long-term, but failed to show the effects of these antiseptics in the skin barrier function and did not evaluate cytokine secretion.

In the present study, we evaluated the effect of antiseptics currently being used in clinic on BASS. Specifically, their effect on cell viability, cytokine secretion patterns, epithelium integrity, and skin barrier integrity were assessed in order to determine the ideal protocol to follow in burn care post-BASS transplantation.

BASS viability assay showed that antiseptic treatment had an effect on cell viability in skin grafts (Figure 2, 3 and Table S1). Cell viability in BASS treated with antiseptics was significantly lower compared to control BASS, showing a dramatic increase of cell death during the evaluation. The only BASS group that showed a similar cell viability rate to control was BASS treated with sodium hypochlorite, which managed to maintain a high cell viability rate throughout the evaluation period (16 days). These results correlate with previous antiseptic toxicity studies performed on hDF and hKT cell types [8,10,11,18].

Regarding BASS cytokine analysis, our studies showed significant differences of cytokine release patterns in BASS treated with antiseptics (Figure 4, Tables S2–S4).

In wound healing, cytokines have the function of coordinating the cellular process, modulating changes in cell growth, differentiation and metabolism in order to restore the damaged tissue and regain functionality [14,19].

TNF-α is a pro-inflammatory cytokine upregulated in the inflammatory phase of wound healing. It is mainly produced by macrophages, but it can also be secreted by a broad variety of cell types, including fibroblasts, keratinocytes, and endothelial cells [20]. TNF-α has a functional duality, engaged both in tissue regeneration and destruction. At low levels, it can promote wound healing by stimulating inflammation and increasing the concentration of macrophage-produced growth factors. At higher levels, TNF-α suppresses the synthesis of ECM and increases the synthesis of metalloproteinases, which degrade the ECM, inhibiting cell migration and collagen deposition [14,20].

FGF is a growth factor family produced by different cell types, such as keratinocytes, fibroblasts, endothelial cells, smooth muscle cells and mast cells [14]. bFGF increases in acute wounds and it has the role to promote angiogenesis, participates in fibroblast proliferation and infiltration, and stimulates epithelial cell migration and proliferation in order to restore the wounded site. Furthermore, it is essential for the synthesis, deposition, and organization of the ECM [14,19].

IL-10 is an anti-inflammatory cytokine that inhibits the production of pro-inflammatory cytokines such as TNF-α, IFN-γ, IL-1β, and IL-6 in macrophages [13,21]. It has a role in anti-scarring epithelial wound repair, attenuating the inflammatory response, regulating the ECM, and fibroblast function and differentiation [13,22]. IL-10 is produced by hematopoietic cells, though some nonhematopoietic cells, such as epithelial cells, are also able to produce IL-10 after injury [13].

In this study, we observed a treatment- and period-dependent alteration of IL-10 and bFGF concentration levels in BASS supernatants. Polyhexanide treatment showed an increase of IL-10 on day 6 of evaluation, which could be explained by a release of IL-10 by hKT as part of the wound healing signaling process, in response to the cell damage caused by the antiseptic treatment [23]. On day 16, however, ethanol, chlorhexidine digluconate, povidone iodine and polyhexanide showed significantly lower levels of IL-10 compared to sodium hypochlorite and control. Combining these results with the cell viability analysis, this decrease of IL-10 level could be explained by the low cell viability in these BASS study groups; the decrease of alive hKT in BASS constructs would stop IL-10 production, thus decreasing IL-10 concentration in the supernatant of BASS.

TNF-α expression is also upregulated in wound injury [23], but no differences were detected throughout the duration of the experiment. This could be explained by the release patterns of these cytokines; TNF-α is a fast-action cytokine and its protein expression reaches its peak 12 h post-injury in hKT. However, TNF-α levels normalize 24 h post-injury due to, among other things, the inhibition of TNF-α expression by IL-10, which reaches its peak concentration 24 h post-injury [21,23]. Thus, the supernatant recollection times in this experiment could not be ideal for TNF-α detection. Another hypothesis would be that the TNF-α concentrations fall under the detection limit of the ELISA kit (30 pg/mL) used for this experiment, hence explaining the lack of differences between study groups throughout the evaluation period. For a more accurate determination of TNF-α and IL-10 concentration, the time of supernatant recollection after treatment should be adjusted.

In the case of bFGF, we observed three concentration spikes during the evaluation time: on ethanol and polyhexanide treatment BASS group on day 9, and povidone iodine treatment BASS on day 16. These spikes correlate to a significant decrease of cell viability in the three cases. Since bFGF stimulates hKT and hDF proliferation in wound healing [14,19], the increase of bFGF in the supernatant may be cell survival signal triggered by the drastic cell death increase. Another description of the bFGF increase would be the liberation of bFGF to the supernatant due to cell lysis [24].

In summary, in terms of cell viability and cytokine expression, sodium hypochlorite was the treatment that held the most similarities to control BASS. Sodium hypochlorite treatment BASS group presented a high cell viability rate and did not show a pro-inflammatory cytokine behavior. Sodium hypochlorite is an antiseptic used in clinic for wound healing treatment and the management of skin disorders [25]. It is a highly-oxidized, pH-neutral solution with a wide antimicrobial spectrum [25,26], achieving a therapeutic activity while maintaining a high skin tolerability and low cytotoxicity.

Therefore, in terms of BASS function and viability, sodium hypochlorite was the least antiseptic treatment option for post-transplantation care in a BASS-based therapy.

As a final evaluation of BASS integrity, the epithelium barrier function of BASS after antiseptic treatment was analyzed. After data analysis, we failed to see any differences in TEWL value between the antiseptic treatment groups and control BASS, even after the application of Vaseline (Figure 5). However, a TEWL value reduction tendency was observed in all study groups after Vaseline application, which was either maintained or showed a minor increase 15 min post-application (Figure 6). Noticeable differences could be seen in BASS thickness and dryness after the dehydration step before TEWL measuring (Figure 8). The dryness of the gel might be the reason for the TEWL value alteration, since treated BASS would have less water to lose, hence obtaining similar TEWL values to BASS that maintain the epithelium barrier function. Another hypothesis as to why there were no differences between antiseptics treatment groups could be that the epithelium might still be too immature to be fully functional, translating into high TEWL values in all study groups.

In either case, TEWL was an ineffective technique to measure epithelial barrier function in BASS with a single epithelial layer. An additional test with a more stratified epithelium needs to be done in order to check epithelium barrier function in BASS. This could be done by growing BASS at an air–liquid interface, which allows the production of a highly stable epithelium by the reproduction of in vivo morphological and biochemical process of the native skin [27,28]. Technical limitations of this technique, such as a limited culture maintenance time and compromised culture viability by excessive thickness of the epithelium, should be considered [27].

## 5. Conclusions

In conclusion, sodium hypochlorite proved to be the least aggressive antiseptic treatment for post-grafting wound care of BASS, due to its low cytotoxicity despite its wide antimicrobial action [29,30]. In BASS constructs, sodium hypochlorite treatment showed a significantly higher cell viability compared to other antiseptic as well as a similar cytokine secretion pattern as BASS control. However, there are still approaches that need to be studied, such as sodium hypochlorite’s possible effect on epithelial barrier function, in order to establish sodium hypochlorite as the standard treatment for BASS wound care.

For a better optimization of BASS technology, a translation from pre-clinical stages to clinical use is needed. The implementation of BASS in clinical trials will help evaluate the safety, behavior and efficacy of BASS grafts, and it will allow the evaluation of the ideal care protocol pre- and post-transplantation for not only in vitro assays, but also in vivo ones.

## Data Availability

Data is contained within the article.

## Supplementary Materials

The following supporting information can be downloaded at: https://www.mdpi.com/article/10.3390/biomedicines10061453/s1, Table S1: Cell viability percentages for each treatment and control at days 6, 9, 13, and 16; *n* = 3, values expressed as mean ± SEM; Table S2: IL-10 concentration levels in BASS supernatants for each treatment and control at days 6, 9, and 16; *n* = 3, values expressed as mean (pg/mL); Table S3: bFGF concentration levels in BASS supernatants for each treatment and control at days 6, 9, and 16; *n* = 3, values expressed as mean (pg/mL); Table S4: TNF-α concentration levels in BASS supernatants for each treatment and control at days 6, 9, and 16; *n* = 3, values expressed as mean (pg/mL).

## Abbreviations

BASS	Bioengineered Autologous Skin Substitutes
bFGF	Basic fibroblast growth factor
CHLO	Chlorhexidine
DFM	Dermal fibroblast medium
DFs	Human dermal fibroblasts
DPBS	Dulbecco’s phosphate-buffered saline
ECM	Extracellular matrix
FBS	Fetal bovine serum
hDF	Human dermal fibroblasts
hKT	Human keratinocytes
IL-10	Interleukin 10
i3T3	Irradiated 3T3 cells
KTM	Keratinocyte medium
TEWL	Trans-epidermal water loss
TNF-α	Tumor necrosis factor alpha
WHO	World Health Organization
