# Supplementary material for: Cytotoxicity, Epidermal Barrier Function and Cytokine Evaluation after Antiseptic Treatment in Bioengineered Autologous Skin Substitute
Source: Biomedicines. 2022 Jun 19;10(6):1453. doi: 10.3390/biomedicines10061453 (PMC9220084; doi:10.3390/biomedicines10061453)
Supplement: Supplementary file 1 [file biomedicines-10-01453-s001.zip › biomedicines-1755670-supplementary.pdf]

Table S1. Cell viability percentages for each treatment and control at days 6, 9, 13, and 16; n = 3, values expressed as mean ± SEM.

Treatment	D6	D9	D13	D16
Ethanol	3.50% ± 1.79	0.69% ± 0.68	0% ± 0	0% ± 0
Chlorhexidine digluconate	71.01% ± 3.21	78.57% ± 8.44	0.20% ± 0.12	0.03% ± 0.03
Sodium hypochlorite	78.16% ± 7.83	96.24% ± 0.64	97.32% ± 1.25	78.83% ± 9.70
Povidone iodine	74.99% ± 6.39	54.71% ± 12.39	25.44% ± 11.84	8.30% ± 3.27
Polyhexanide	11.09% ± 3.21	0.11% ± 0.11	0.05% ± 0.04	0.90% ± 0.79
Control	90.48% ± 6.77	97.18% ± 2.31	90.15% ± 4.05	87.02% ± 2.06

Table S2. IL-10 concentration levels in BASS supernatants for each treatment and control at days 6, 9, and 16; n = 3, values expressed as mean (pg/mL).

Treatment	D6	D9	D16
Ethanol	26.13 ± 1.19	12.06 ± 2.33	13.21 ± 0.28
Chlorhexidine digluconate	22.13 ± 0.59	16.33 ± 0.56	12.83 ± 0.80
Sodium hypochlorite	25.60 ± 1.15	17.03 ± 0.56	22.58 ± 0.84
Povidone iodine	22.41 ± 0.31	15.64 ± 1.74	14.42 ± 0.73
Polyhexanide	29.98 ± 3.72	17.38 ± 1.88	15.64 ± 0.56
Control	23.49 ± 2.29	16.78 ± 0.79	21.82 ± 1.11

Table S3. bFGF concentration levels in BASS supernatants for each treatment and control at days 6, 9, and 16; n = 3, values expressed as mean (pg/mL).

Treatment	D6	D9	D16
Ethanol	963.00 ± 57.50	5336.75 ± 336.25	565.50 ± 5.00
Chlorhexidine digluconate	888.00 ± 47.50	605.50 ± 10.00	871.75 ± 18.75
Sodium hypochlorite	743.00 ± 100.00	720.50 ± 22.50	1003.00 ± 125.00
Povidone iodine	760.50 ± 80.00	700.50 ± 5.00	2841.00 ± 55.50
Polyhexanide	748.00 ± 67.50	1494.25 ± 101.25	583.00 ± 2.50
Control	801.75 ± 21.25	670.50 ± 15.00	711.75 ± 13.75

Table S4. TNF-α concentration levels in BASS supernatants for each treatment and control at days 6, 9, and 16; n = 3, values expressed as mean (pg/mL).

Treatment	D6	D9	D16
Ethanol	94.71 ± 4.79	85.54 ± 3.54	93.25 ± 7.92
Chlorhexidine digluconate	77.21 ± 0.21	81.79 ± 6.04	82.42 ± 7.92
Sodium hypochlorite	74.50 ± 5.83	83.25 ± 2.92	82.42 ± 12.09
Povidone iodine	77.00 ± 2.50	87.21 ± 10.63	85.33 ± 4.17
Polyhexanide	101.38 ± 18.13	77.63 ± 4.79	97.83 ± 5.00
Control	81.38 ± 11.05	79.08 ± 1.25	101.58 ± 2.09
